# Pre-test of questions on health-related resource use and expenditure, using behaviour coding and cognitive interviewing techniques

**DOI:** 10.1186/1472-6963-12-303

**Published:** 2012-09-06

**Authors:** Nadja Chernyak, Corinna Ernsting, Andrea Icks

**Affiliations:** 1 Department of Public Health, Faculty of Medicine, Heinrich-Heine University Düsseldorf, Moorenstrasse 5, 40225, Düsseldorf, Germany; 2 German Diabetes Center, Institute of Biometrics and Epidemiology, Auf’m Hennekamp 65, 40225, Düsseldorf, Germany

**Keywords:** Self-reported health care utilisation, Survey research, Questionnaire design, Behaviour coding, Cognitive interviewing

## Abstract

**Background:**

Validated instruments collecting data on health-related resource use are lacking, but required, for example, to investigate predictors of healthcare use or for health economic evaluation.

The objective of the study was to develop, test and refine a questionnaire collecting data on health-related resource use and expenditure in patients with diabetes.

**Methods:**

The questionnaire was tested in 43 patients with diabetes mellitus types 1 and 2 in Germany. Response behaviour suggestive of problems with questions (item non-response, request for clarification, comments, inadequate answer, “don’t know”) was systematically registered. Cognitive interviews focusing on information retrieval and comprehension problems were carried out.

**Results:**

Many participants had difficulties answering questions pertaining to frequency of visits to the general practitioner (26%), time spent receiving healthcare services (39%), regular medication currently taken (35%) and out of pocket expenditure on medication (42%). These difficulties seem to result mainly from poor memory. A number of comprehension problems were established and relevant questions were revised accordingly.

**Conclusion:**

The questionnaire on health-related resource use and expenditure for use in diabetes research in Germany was developed and refined after careful testing. Ideally, the questionnaire should be externally validated for different modes of administration and recall periods within a variety of populations.

## Background

Data on health care utilisation (e.g. hospitalisations, use of outpatient health services, medication use) is required for several reasons. For example, to analyse service use patterns, to identify “underuse” or “overuse” of health care services, to assess health care needs or health-related costs. Data on health care utilisation may be obtained from various sources: health insurance claims, disease registries, provider records, patient self-report, and expert opinion.

In principle, if detailed information is required, provider records may be a better source of utilisation data than burdening patients with a detailed data collection procedure by means of a diary, a written questionnaire or an interview [1]. In practice, however, provider records are often difficult to access or retrieve, because (i) it may be necessary to contact many different providers and (ii) the patient’s consent may be required but not forthcoming. Often, the same limitations to data availability also apply to health insurance data, in particular, if linking health insurance data to other data sources (e.g. data from a clinical trial or a survey) is necessary. Accuracy and completeness of administrative data are also a concern when providers are in a capitation fee system and have little financial incentive to record diagnoses and services accurately [2]. Moreover, information on utilisation of health services not covered by the health insurance and on the non-medical resource-use (e.g. patient’s or caregiver’s time) is usually not available from health insurance or provider records.

Consequently, researchers as well as public policymakers often have to rely – at least in part – on self-reported health care utilisation. Considerable work is usually undertaken in individual projects to develop self-report tools (i.e. questionnaires or diaries) collecting data on health-related resource use. Yet these tools and the results of their validation are seldom published and there seems to be a tendency to develop new questionnaires for new studies [3,4]. Bertoldi et al. [3] conducted a review of the methodologies used in household surveys on medication use (61 studies published between January 1995 and June 2008 were included in the review). They showed that 70% of the studies did not publish the questions used to assess medication use; and 93.4% of the studies provided no information on the validity of the questionnaire employed to collect data on medication use. If methods of assessment of health care utilisation are not appropriate, results may be invalid. For example, Bhandari et al. [2] found that physician visits have been underreported by up to 50%, depending on the recall period covered by a questionnaire. Hence, publication of questionnaires, as well as results of their validation can contribute to the standardisation of data collection methods and comparability of results across different studies.

To analyse the current situation in Germany, we conducted a systematic search in MEDLINE and the Cochrane library (Method studies) to identify published validated or at least standardised self-report instruments appropriate to collect healthcare utilisation data in Germany (search strategy, inclusion and exclusion criteria and the list of identified instruments can be obtained from the first author). Despite extensive research on health care utilisation and costs per se, a validated or at least standardised set of questions on health care utilisation in German language was not identified by this search. Thus we developed, tested and subsequently refined a German questionnaire on health-related resource-use and expenditure. Patients with diabetes constituted our specific target group. However, it is often difficult to attribute resource utilisation and cost to a specific disease. Therefore, in addition to a disease-specific module, the instrument includes a generic module collecting data on a broad range of resource use categories. Hence the questionnaire can be used both in patients with diabetes and – omitting a diabetes-specific profile – also in the general population or in other patient populations. The questionnaire can be used in cross-sectional studies and for longitudinal data collection, for example, in conjunction with a clinical trial.

## Methods

### Development of a questionnaire on health-related resource use and expenditure for use in diabetes research

Potentially relevant questions on health care utilisation were identified by reviewing German questionnaires obtained via Internet and personal communication with different research groups – mainly, instruments developed for large scale German health surveys and epidemiologic studies, by Robert Koch institute, by TNS Health care for the Health Care Monitor project, as well as instruments developed for the Cooperative Health Research in the Region of Augsburg (KORA) study [5] and the Heinz Nixdorf Recall study [6]. Alternative approaches to asking questions covering similar content were grouped together, and a set of questions on general health care utilisation (primary care visits, visits to emergency departments, visits to specialists, hospital stays, and other therapies or paramedical care) was assembled either by adopting wording from those candidate questions directly or by adapting them to fit a question form seemingly more appropriate to the new instrument.

Specific diabetes-related questions were elaborated in close cooperation with clinical experts in the field of diabetology from two large specialized diabetes centres in Germany – the German Diabetes Centre in Düsseldorf and the research institute of the Diabetes Academy Mergentheim.

Particular attention was paid to the development of questions regarding medication use (medication name, strength, prescribed frequency, and duration of use). Items were developed that captured (i) current use of diabetes-specific medication, (ii) changes in diabetes-specific medication profile within a defined reference period, i.e. changes with regard to the number of medications taken, the dosage level for one or more medications, and the pharmacologic class of the medications being taken; (iii) current use of regularly-taken medications for other conditions and (iv) occasionally-taken medication within a defined reference period.

The initial questionnaire collected data on (i) a wide range of health care services utilisation during a specified recall period (number of visits to the general practitioner, including home visits, number of visits to emergency room or departments, number of outpatient visits to various specialists and therapists, utilisation of hospital outpatient services, diagnostic tests and procedures carried out ambulatorily, hospital inpatient admissions and their duration; (ii) time spent obtaining these healthcare services, (iii) use of diabetes-specific and other medication; (iv) out-of-pocket expenditure on medication; (v) comorbidity; (vi) disability days and days off work; and (vii) unpaid or paid help received by the patient because of a limited ability to do household chores (the time for which help was needed and corresponding cost, if applicable); (viii) other variables possibly required to analyse the data (e.g. participation in disease management programmes and employment status).

### Test of the questionnaire

#### Study setting and participants

To test the developed questionnaire, patients with diabetes mellitus were recruited from a general practitioner’s office and an outpatient centre specialising in diabetes treatment in two cities in North Rhine-Westfalia in Germany. We aimed to recruit respondents covering the range of individuals with diabetes, e.g. those with type 1 and type 2 diabetes, younger and older patients and those with different socioeconomic status, who may all be sampled in future surveys or recruited to RCTs, that is, a sampling approach aiming to explore diversity by maximising variance was applied. The questionnaire was tested by a combination of two techniques – behaviour coding and cognitive interviewing (see below). Since it is recommended that behaviour coding studies contain at least 30 respondents [7], we recruited more patients than would be needed for cognitive interviewing alone.

#### Testing procedure

As a means of systematically identifying questions which needs revision, behaviour coding [8] was carried out. A standardized behaviour coding form was employed to document problem indicators, i.e. behaviour suggestive of problems with a particular question. The following problem indicators were registered for each question: (i) request for clarification; (ii) answer with comments, i.e. answer appears to meet question objective, but comments indicate uncertainty, misunderstanding, etc.; (iii) inadequate answer, i.e. answer that does not meet question objective; (iv) “don’t know” answer; (v) item non-response; and (vi) “no improvement after probing” if the answer was inadequate even after feedback from the interviewer.

In order to explain problems registered by the behaviour coding and to reveal problems not evident in response behaviour such as silent misinterpretation, cognitive interviewing [7] was added to the testing procedure. To this end, both scripted and unscripted probes were used. Scripted probes aimed to (i) provide some standardisation of analyses across interviews and (ii) to ensure that the survey questions of greatest concern were probed appropriately within the limited cognitive interview time. Three various types of scripted probes were employed: (i) c*omprehension probes* asking respondents to explain their understanding of particular concepts or terms; (ii) *cursive retrieval probes* asking them to explain how respondents arrived at an answer and (iii) cursive asking respondents to evaluate the degree of confidence in their answers. Unscripted probes were usually applied if the respondent gave an inadequate answer or if item non-response occurred. The protocol concluded with a series of general questions allowing the respondent to provide additional feedback on particular items or the questionnaire as a whole.

The questionnaire was tested both as self-administered and as an interviewer-administered tool. In the self-administration group, participants were instructed to report any difficulties or problems with questions while completing the questionnaire, in order to enable behaviour coding. Problem indicators “inadequate answer“ and “item non-response” (i.e. missing) were assigned during a follow-up interview and by a subsequent analysis of the completed questionnaires. In this group, a retrospective approach to cognitive interviews was adopted. Once participants had completed the entire questionnaire, the interviewer went back through it, asking follow-up questions (probes).

In the interviewer-administered group, behaviour was coded during the interview. A concurrent approach to cognitive interviews was implemented, i.e. the interviewer read the survey questions aloud and probed immediately after the respondent had answered a particular question. The concurrent strategy aimed to avoid retrospection problems, which might occur when probing is carried out at the end of the questionnaire. However, concurrent probing can influence responses to subsequent questions and it was important to take this possibility into account by implementing retrospective probing in the self-administration group.

The study was conducted in September – October 2010 by two researchers acting as interviewer (CE) and observer (NC). Both received training in cognitive interviewing techniques prior to conducting interviews. Test-interviews supervised by a psychologist were conducted in the diabetes outpatient department of the University Clinical Centre in Düsseldorf. Test interviews were also used to test and modify problem indicators for behaviour coding and to finalise the cognitive interview protocol.

### Analysis

The interviews were tape-recorded with the permission of the respondents. Both researchers (interviewer and observer) listened to the audiotapes and independently performed behaviour coding from the tape-recordings. Behaviour coding performed during the interviews was also reviewed. Disagreement with regard to behaviour coding was solved by consensus between the researchers. Coding summaries reflecting the relative frequency of problem indicators across all interviews were produced for each question. According to a guideline cut-off point proposed in the literature [8,9], questions were classified as problematic if 15% or more of responders had problem(s) with a question, i.e. at least one problem indicator was assigned to the question in 15% or more of the interviews.

Qualitative analysis of cognitive interviews was also performed by both researchers. Summaries highlighting problems pertaining to particular questions were created. These summaries served as a basis for revision of the questionnaire.

### Ethical considerations

The study was approved by the Ethics Committee of the Medical Faculty, University of Düsseldorf, on April 30, 2010 (Reference number: 3370). Informed consent, which is required in accordance with the Declaration of Helsinki, was obtained from each individual participating in the study.

## Results

Forty-three patients participated in the study, 10 patients with diabetes mellitus type 1 and 33 with diabetes mellitus type 2. Seventeen of the respondents were male and 26 female. Respondent age ranged from 21 to 83 years (mean = 60.0). Some characteristics of respondents are presented in Table 1. The questionnaire was tested as self-administered and as an interviewer-administered tool in 19 and 24 patients respectively.

**Table 1:** Respondent characteristics

**Respondent characteristics**	**N**
**Years of schooling**	
No schooling	3
≤10 years	29
12-13 years	7
Not specified/ unclear	4
**Employment status**	
Working full-time	12
Working part-time	5
Unemployed	4
At home	3
Retired	19

### Response behaviour

Questions in which at least one problem indicator occurred in 15% or more of interviews are shown in Table 2. “Item non-response”, “inadequate answer” and “don’t know” indicate serious problems likely to influence the quality of resulting data. High levels of these problem indicators were registered for questions on the number of visits to the general practitioner in the last 6 months, the number of visits to specialists in the last 6 months, time spent on receiving health care services in the last 6 months, currently-taken regular medication, and out-of-pocket expenditure on medication in the last 6 months.

**Table 2:** Distribution of problem indicator levels for the questions which caused difficulties to 15% or more of respondents

***Questions***	**Problem Indicators**					
	**Item non response/ missing**	**Inadequate answer**	**Request for clarification**	**Answer with comments**	**Do not know**	**No improvement by probing**
1. In the last 6 months, have you seen your primary care physician or have you had to ask for a house call? If Yes, please specify the number of contacts?	18	19	9	8	6	11
2. In the last 6 months, have you visited the emergency room or a medical emergency service or something similar due to an emergency? If Yes, please specify the number of contacts?	2	14	1	1	0	3
3. In the last 6 months, have you seen any of the following physicians having their own practice (a list provided)? If Yes, please specify the number of contacts?	10	13	7	2	2	5
4. Please provide an estimate of how much time you have spent on all your outpatient doctor visits in the last 6 months. Please also consider travel time to and from physicians and time spent waiting.	16	4	5	10	8	17
5. In the last 6 months, have you had any of the following special medical tests (a list provided)? Please check all that apply. If Yes, please specify how many times?	8	3	2	4	3	3
6. In the last 6 months, have you gone to see a physical therapist, naturopath, or other therapists (a list provided)? If Yes, please specify the number of contacts.	2	8	1	4	0	1
7. In the last 6 months, have there been any treatment changes with regard to your diabetes treatment? If Yes, please check all that apply (for each treatment a list of possible changes, i.e. newly prescribed, discontinued, dose reduced, dose increased was provided and participants were asked when the changes occurred).	5	10	4	5	1	0
8. If you are treated with blood-sugar lowering tablets at present, please provide the exact medication name and the daily dose.	9	3	1	0	1	4
9. If you are treated with insulin at present, please indicate how you administer insulin, the exact insulin product name and units per day.	7	1	1	1	0	1
10. Please indicate which medications you REGULARLY take in addition to your diabetes therapy at present. Please specify exact medication name, form of administration (tablets, liquid, etc.) and daily dose.	21	12	7	3	5	15
11. Are there any other medications that you have been taking AS NEEDED in the last 6 months? If Yes, please specify exact medication name, form of administration (tablets, liquid, etc.), daily dose and frequency of use in the last 6 months.	11	3	1	1	1	8
12. In the last 6 months, how much have you paid for all of your medications (including expenses for prescription fees)? If you are not able to indicate the exact amount, please provide an estimate.	14	10	10	11	8	18

Probing substantially improved initial results for many questions (see last column of Table 2). The question as to the number of visits to specialists is rather typical in this regard. Initially, there was a high proportion of missing and inadequate answers. Many participants did not state the number of visits in the last 6 months, as required by the question, but rather answered with a rate per time period (e.g. 1 visit every 3 months). In such cases probing usually resulted in the adequate answer, that is, the number of visits. However, many participants were not able to give an adequate answer with regard to frequency of visits to the general practitioner (26%), time spent receiving healthcare services (39%), regular medication currently taken (35%) and out-of-pocket expenditure on medication (42%), even after probing.

### Information retrieval problems

Item non-response, inadequate answer, or “don’t know” answer may be associated with information retrieval problems. Indeed, difficulty in estimating time spent receiving health care and out-of-pocket expenditure on medication evident from behaviour coding was confirmed by the confidence rating – 28% of respondents to these questions admitted that it was a rough estimate.

Most respondents were unable to explain how they arrived at their answers regarding the number of visits to the general practitioner and to various medical specialists in the last 6 months, i.e. whether they counted single visits, made an estimate, etc. Interestingly, when asked, many respondents were very confident about their answers, even though their response behaviour suggested otherwise. This is illustrated in Table 3.

**Table 3:** Confidence rating* regarding the number of visits to the GP and to various physicians

**Confidence rating**	**Number of visits to GP**	**Number of visits to specialists**
Exact	25	37
Not very precise	8	0
Rough estimate	7	0
Not conducted	3	6

*How difficult was it for you to recall the number of visits in the last 6 months? Do you think the number of visits you gave is exact, not very precise or a rough estimate?

### Comprehension problems

In sum, the following comprehension problems were identified:

 • 19 respondents did not distinguish between the general practitioner (primary care doctor), internist and diabetologist, and tended to count the visits to these physicians twice, i.e. they first mentioned the visits in response to the question about the visits to the GP, then mentioned them again in response to the question about visits to the specialist (the question includes a list of various medical specialists).

 • For some respondents it was not clear that the question about the number of visits to specialists in the last 6 months explicitly asks about the frequency of visits to the specialists working in the ambulatory sector, not in the hospital. 

 • Most respondents were not able to clearly differentiate between a psychiatrist, a psychotherapist, and a psychologist. Twenty participants knew that there is a distinction, but could not correctly explain it. Some participants also mentioned “neurologist” here. Twenty-two respondents did not even make a distinction.

 • Some respondents mentioned emergency care obtained in the hospital in response to the question about ambulatory healthcare services provided in hospital, although the question explicitly instructed them not to include emergency care.

 • Others mentioned overnight hospital stays in response to the question asking about emergency care visits.

 • Many respondents found the question aiming to capture changes in diabetes-specific therapy (medication, insulin) in the previ**o**us 6 months either unclear or difficult.

 •The term “medication” was not understood consistently by all respondents. Accordingly, when asked about their out-of-pocket expenditure on medication, some respondents considered only prescription medication, but not over-the-counter medication.

### Revision of questions

Revisions were mainly undertaken to overcome comprehension difficulties encountered in cognitive interviews. We revised questions about visits to primary care and other physicians, ambulatory care provided in hospital, emergency care, medication, diabetes-specific therapy and time receiving health care. Modifications included changes in wording, the introduction of additional instructions, as well as the re-arrangement and splitting of questions (see Table 4).

**Table 4:** Modification of the questionnaire

**Comprehension problem**	**Modification of the questionnaire**
Respondents did not distinguish between the general practitioner, internist and diabetologist.	Additional instructions introduced
Respondents did not distinguish between specialists working in the ambulatory sector and in the hospital.	Wording changed
Respondents did not distinguish between a psychiatrist, a psychotherapist, and a psychologist.	Explanations added
Respondents did not distinguish between emergency care and ambulatory healthcare services provided in hospital.	Wording changed
Respondents mentioned overnight hospital stays in response to the question asking about emergency care visits.	Wording changed
Respondents found the question with regard to changes in diabetes-specific therapy (medication, insulin) in the previous 6 months unclear or difficult.	Two separate questions referring to medication and insulin formulated
Question about out-of-pocket expenditure on medication was not understood in a consistent way.	Two separate questions referring to the prescription medication and over-the-counter medication formulated

Because questions capturing medication use were shown to be particularly difficult, as well as very time-consuming, a computer-assisted version of these questions was developed to make data collection more efficient. The items are displayed to the interviewer on a computer screen; the interviewer reads the questions to the respondent and enters their responses directly into the electronic database. Moreover, if the medication packages are available at the time of the interview, barcodes can be scanned. This procedure, which has already been used in several surveys [5], allows classification of the medications according to the Anatomical Therapeutic Chemical (ATC) Classification System and obviates the need to collect the information on the name, strength and form of the medication. In this case, participants only have to report the frequency of medication use and the duration of administration. The revised version of the questionnaire is provided in the Additional files 1 and 2.^i^

## Discussion

The questionnaire was tested by a combination of two established techniques – behaviour coding and cognitive interviewing [8-14]. Behaviour coding shows how often response behaviours defined a-priori, and suggestive of difficulties with a particular question, occur across interviews. The strength of this technique is its systematic and quantitative nature, allowing comparison across questions. However, it is somewhat arbitrary what rate of a problem indicator should be considered high enough to constitute a problem with the question. We used the guideline cut-off point of 15% proposed in the literature [8,9].

Cognitive interviews were conducted to identify possible reasons for any problems observed and to allow for more qualitative analysis of difficulties with questions. Results of cognitive interviews suggest that in many cases difficulties reflected in response behaviour can be explained by inability to retrieve the information required, for example, due to recall problems. Often, extensive probing was needed to obtain an answer. Hence it seems that information retrieval may be improved by interviewer-administration or by additional probes following written administration of questions. However, this challenges the possibility to collect valid health care utilisation data by self-administered questionnaires.

Cognitive interviews can indicate the existence of problems with questions, for example, difficulties pertaining to a particular recall period or to the mode of administration. However, they cannot provide quantitative information on the quality of self-reported data. To obtain this type of information, studies comparing self-reported data to data from alternative sources are required.

As already mentioned in the introduction, it is often difficult to attribute resource utilisation and cost to a specific disease. A pragmatic approach to handling the issue of cost attribution may be to apply a generic questionnaire collecting data on a broad range of resource-use categories and subsequently to attempt to determine their attribution. To enable meaningful analysis following this approach, detailed data on comorbidity should also be collected. Hence more research with regard to validity of self-reported comorbidity is needed.

## Conclusion

We developed the questionnaire on health-related resource use and expenditure for use in diabetes research in Germany. In addition to a disease-specific module, the instrument includes a generic module collecting data on a broad range of resource-use categories. Hence the questionnaire can be used both in diabetic patients and – omitting a diabetic-specific profile – also in the general population or other patient populations.

Efforts to further standardise the questionnaire should be based on validation studies. Ideally, the questions should be externally validated according to a range of criteria (e.g. for different recall periods and modes of administration) and within a variety of populations, in order to meaningfully interpret health care utilisation reported in single studies and to compare findings across studies. The questionnaire is currently being validated for two different recall periods applying an experimental design.

## Endnotes

^i^The English version of the questionnaire presented in the Additional file 2 is a translation of the refined German version presented in the Additional file 1. The translational process used forward and backward translation techniques. However, further adaptation of the English version would be required to take the peculiarities of the service provision in other countries into account.

## Competing interests

The authors declare that they have no competing interests.

## Authors’ contributions

NC and CE participated in the design of the study, carried it out and performed the analysis (both authors contributed equally to this work). NC drafted the manuscript. AI conceived the study and participated in its coordination. All co-authors read, edited, and approved the final manuscript.

## Pre-publication history

The pre-publication history for this paper can be accessed here:

http://www.biomedcentral.com/1472-6963/12/303/prepub

## Supplementary Material

Additional file 1 Revised questionnaire (German version). Click here for file

Additional file 2 Revised questionnaire (English translation). Click here for file
