# Supplementary material for: Pre-test of questions on health-related resource use and expenditure, using behaviour coding and cognitive interviewing techniques
Source: BMC Health Serv Res. 2012 Sep 6;12:303. doi: 10.1186/1472-6963-12-303 (PMC3470966; doi:10.1186/1472-6963-12-303)
Supplement: Additional file 1 — Revised questionnaire (German version). [file 1472-6963-12-303-S1.pdf]

Probanden-ID: _______________ Datum: ___________ Interviewer-ID: __________

Fragebogen

zu Gesundheit und medizinischer Versorgung

Herzlichen Dank, dass Sie an dieser Befragung teilnehmen!

DDZ
Deutsches Diabetes-Zentrum

HEINRICH HEINE UNIVERSITÄT DÜSSELDORF

pronova BKK
PARTNER FÜR IHRE GESUNDHEIT

Probanden-ID: ________________ Datum: ____________ Interviewer-ID: ___________

Diabetes, andere Erkrankungen und gesundheitliche Probleme

1. Welchen Diabetes-Typ haben Sie?

Typ-1 |__|

Typ-2 („Altersdiabetes“) |__|

Anderer |__|

und zwar: ____________________

weiß ich nicht |__|

2. Wann wurde der Diabetes bei Ihnen diagnostiziert?

vor _______ Jahren oder Jahresangabe: __________

vor _______ Monaten (wenn Diagnose weniger als 1 Jahr zurückliegt)

weiß ich nicht |__|

3. Auf den folgenden Seiten finden Sie eine Auflistung verschiedener Erkrankungen. Bitte geben Sie an, welche dieser Erkrankungen Sie haben bzw. in den letzten 12 Monaten hatten. Gemeint sind Erkrankungen, die Ihre Ärztin/ Ihr Arzt bei Ihnen festgestellt hat.

Falls Sie in der ersten Spalte „Nein“ ankreuzen, fahren Sie bitte direkt mit der nächsten Erkrankung in der darunterliegenden Zeile fort. Falls Sie „Ja“ ankreuzen, geben Sie bitte an, ob Sie sich aufgrund dieser Erkrankung in (ärztlicher) Behandlung befinden und ob diese Erkrankung Sie in der Ausübung Ihrer täglichen Aktivitäten (Arbeit bzw. Freizeit) beeinträchtigt. Am Ende der Liste haben Sie die Möglichkeit, weitere Erkrankungen zu ergänzen, die bislang nicht aufgeführt wurden.

**Erkrankung**	**Ist die Erkrankung von Ihrer Ärztin/ Ihrem Arzt festgestellt worden?**	**Befinden Sie sich deshalb in (ärztlicher) Behandlung?**	**Wie stark beeinträchtigt Sie diese Erkrankung in der Ausübung Ihrer täglichen Aktivitäten (Arbeit bzw. Freizeit)?**
Hypertonie (Bluthochdruck)	|__| Ja |__| Nein	|__| Ja |__| Nein	|__| Gar nicht |__| Ein wenig |__| Mittelmäßig |__| Stark |__| Sehr stark
Periphere arterielle Verschlusskrankheit („Schaufensterkrankheit“: Schmerzen in den Beinen oder Waden beim Gehen, weshalb Sie stehen bleiben müssen, damit der Schmerz nachlässt)	|__| Ja |__| Nein	|__| Ja |__| Nein	|__| Gar nicht |__| Ein wenig |__| Mittelmäßig |__| Stark |__| Sehr stark
Durchblutungsstörungen am Herzen (Angina pectoris)	|__| Ja |__| Nein	|__| Ja |__| Nein	|__| Gar nicht |__| Ein wenig |__| Mittelmäßig |__| Stark |__| Sehr stark
Herzinfarkt	|__| Ja |__| Nein	|__| Ja |__| Nein	|__| Gar nicht |__| Ein wenig |__| Mittelmäßig |__| Stark |__| Sehr stark
Herzinsuffizienz (Herzschwäche)	|__| Ja |__| Nein	|__| Ja |__| Nein	|__| Gar nicht |__| Ein wenig |__| Mittelmäßig |__| Stark |__| Sehr stark
Durchblutungsstörung des Gehirns	|__| Ja |__| Nein	|__| Ja |__| Nein	|__| Gar nicht |__| Ein wenig |__| Mittelmäßig |__| Stark |__| Sehr stark
Schlaganfall	|__| Ja |__| Nein	|__| Ja |__| Nein	|__| Gar nicht |__| Ein wenig |__| Mittelmäßig |__| Stark |__| Sehr stark
TIA (vorübergehende Durchblutungsstörung des Gehirns mit schlaganfallähnlichen Symptomen)	|__| Ja |__| Nein	|__| Ja |__| Nein	|__| Gar nicht |__| Ein wenig |__| Mittelmäßig |__| Stark |__| Sehr stark
Erkrankungen der Augen (z.B. Schäden am Augenhintergrund, Katarakt)	|__| Ja |__| Nein	|__| Ja |__| Nein	|__| Gar nicht |__| Ein wenig |__| Mittelmäßig |__| Stark |__| Sehr stark
Erkrankungen der Nerven an den Beinen bzw. Füßen (z.B. Brennen, Kribbeln oder Taubheitsgefühl)	|__| Ja |__| Nein	|__| Ja |__| Nein	|__| Gar nicht |__| Ein wenig |__| Mittelmäßig |__| Stark |__| Sehr stark

Probanden-ID: ____________

Probanden-ID: ______

**Erkrankung**	**Ist die Erkrankung von Ihrer Ärztin/ Ihrem Arzt festgestellt worden?**	**Befinden Sie sich deshalb in (ärztlicher) Behandlung?**	**Wie stark beeinträchtigt Sie diese Erkrankung in der Ausübung Ihrer täglichen Aktivitäten (Arbeit bzw. Freizeit)?**
Entzündungen, Geschwüre oder Wunden an den Füßen, die schlecht heilen	|__| Ja |__| Nein	|__| Ja |__| Nein	|__| Gar nicht |__| Ein wenig |__| Mittelmäßig |__| Stark |__| Sehr stark
Amputation an Füßen/ Beinen	|__| Ja |__| Nein	|__| Ja |__| Nein	|__| Gar nicht |__| Ein wenig |__| Mittelmäßig |__| Stark |__| Sehr stark
Erkrankungen der Niere (z.B. Eiweißausscheidung im Urin)	|__| Ja |__| Nein	|__| Ja |__| Nein	|__| Gar nicht |__| Ein wenig |__| Mittelmäßig |__| Stark |__| Sehr stark
Dialysepflichtigkeit (Blutwäsche)	|__| Ja |__| Nein	|__| Ja |__| Nein	|__| Gar nicht |__| Ein wenig |__| Mittelmäßig |__| Stark |__| Sehr stark
Krebserkrankung (bösartiger Tumor)	|__| Ja |__| Nein	|__| Ja |__| Nein	|__| Gar nicht |__| Ein wenig |__| Mittelmäßig |__| Stark |__| Sehr stark
Schilddrüsenerkrankung	|__| Ja |__| Nein	|__| Ja |__| Nein	|__| Gar nicht |__| Ein wenig |__| Mittelmäßig |__| Stark |__| Sehr stark
Gicht	|__| Ja |__| Nein	|__| Ja |__| Nein	|__| Gar nicht |__| Ein wenig |__| Mittelmäßig |__| Stark |__| Sehr stark
Chronische (andauernde) Rückenschmerzen	|__| Ja |__| Nein	|__| Ja |__| Nein	|__| Gar nicht |__| Ein wenig |__| Mittelmäßig |__| Stark |__| Sehr stark
Entzündliche Gelenk- oder Wirbelsäulenerkrankung (z.B. Arthritis)	|__| Ja |__| Nein	|__| Ja |__| Nein	|__| Gar nicht |__| Ein wenig |__| Mittelmäßig |__| Stark |__| Sehr stark
Sonstige Gelenk- oder Wirbelsäulenerkrankung	|__| Ja |__| Nein	|__| Ja |__| Nein	|__| Gar nicht |__| Ein wenig |__| Mittelmäßig |__| Stark |__| Sehr stark
Magen- oder Zwölffingerdarmgeschwür oder chronische Magenschleimhautentzündung	|__| Ja |__| Nein	|__| Ja |__| Nein	|__| Gar nicht |__| Ein wenig |__| Mittelmäßig |__| Stark |__| Sehr stark
Entzündliche Darmerkrankung (z.B. Colitis Ulcerosa, Morbus Crohn)	|__| Ja |__| Nein	|__| Ja |__| Nein	|__| Gar nicht |__| Ein wenig |__| Mittelmäßig |__| Stark |__| Sehr stark
Sonstige Darmerkrankung	|__| Ja |__| Nein	|__| Ja |__| Nein	|__| Gar nicht |__| Ein wenig |__| Mittelmäßig |__| Stark |__| Sehr stark

<table>
<tr><th>Erkrankung</th><th>Ist die Erkran-kung von Ihrer Ärztin/ Ihrem Arzt festgestellt worden?</th><th>Befinden Sie sich deshalb in (ärztlicher) Behandlung?</th><th>Wie stark beeinträchtigt Sie diese Erkrankung in der Ausübung Ihrer täglichen Aktivitäten (Arbeit bzw. Freizeit)?</th></tr>
<tr><td>Gallensteine</td><td>|__| Ja |__| Nein</td><td>|__| Ja |__| Nein</td><td>|__| Gar nicht |__| Ein wenig |__| Mittelmäßig |__| Stark |__| Sehr stark</td></tr>
<tr><td>Häufige Harnwegsinfektionen (Blasenentzündung)</td><td>|__| Ja |__| Nein</td><td>|__| Ja |__| Nein</td><td>|__| Gar nicht |__| Ein wenig |__| Mittelmäßig |__| Stark |__| Sehr stark</td></tr>
<tr><td>Chronische Leberentzündung (Hepatitis)</td><td>|__| Ja |__| Nein</td><td>|__| Ja |__| Nein</td><td>|__| Gar nicht |__| Ein wenig |__| Mittelmäßig |__| Stark |__| Sehr stark</td></tr>
<tr><td>Allergie(n), Heuschnupfen</td><td>|__| Ja |__| Nein</td><td>|__| Ja |__| Nein</td><td>|__| Gar nicht |__| Ein wenig |__| Mittelmäßig |__| Stark |__| Sehr stark</td></tr>
<tr><td>Asthma bronchiale</td><td>|__| Ja |__| Nein</td><td>|__| Ja |__| Nein</td><td>|__| Gar nicht |__| Ein wenig |__| Mittelmäßig |__| Stark |__| Sehr stark</td></tr>
<tr><td>Chronische Bronchitis oder chronisch obstruktive Lungenerkrankung (COPD)</td><td>|__| Ja |__| Nein</td><td>|__| Ja |__| Nein</td><td>|__| Gar nicht |__| Ein wenig |__| Mittelmäßig |__| Stark |__| Sehr stark</td></tr>
<tr><td>Blutarmut (Anämie)</td><td>|__| Ja |__| Nein</td><td>|__| Ja |__| Nein</td><td>|__| Gar nicht |__| Ein wenig |__| Mittelmäßig |__| Stark |__| Sehr stark</td></tr>
<tr><td>Chronische Hautkrankheiten (z.B. Neurodermitis, Schuppenflechte/ Psoriasis)</td><td>|__| Ja |__| Nein</td><td>|__| Ja |__| Nein</td><td>|__| Gar nicht |__| Ein wenig |__| Mittelmäßig |__| Stark |__| Sehr stark</td></tr>
<tr><td>Migräne</td><td>|__| Ja |__| Nein</td><td>|__| Ja |__| Nein</td><td>|__| Gar nicht |__| Ein wenig |__| Mittelmäßig |__| Stark |__| Sehr stark</td></tr>
<tr><td>Epilepsie</td><td>|__| Ja |__| Nein</td><td>|__| Ja |__| Nein</td><td>|__| Gar nicht |__| Ein wenig |__| Mittelmäßig |__| Stark |__| Sehr stark</td></tr>
<tr><td>Parkinson-Krankheit</td><td>|__| Ja |__| Nein</td><td>|__| Ja |__| Nein</td><td>|__| Gar nicht |__| Ein wenig |__| Mittelmäßig |__| Stark |__| Sehr stark</td></tr>
<tr><td>Depression</td><td>|__| Ja |__| Nein</td><td>|__| Ja |__| Nein</td><td>|__| Gar nicht |__| Ein wenig |__| Mittelmäßig |__| Stark |__| Sehr stark</td></tr>
<tr><td>Sonstige Erkrankung, und zwar:
______________________________</td><td>|__| Ja |__| Nein</td><td>|__| Ja |__| Nein</td><td>|__| Gar nicht |__| Ein wenig |__| Mittelmäßig |__| Stark |__| Sehr stark</td></tr>
<tr><td>Sonstige Erkrankung, und zwar:
______________________________</td><td>|__| Ja |__| Nein</td><td>|__| Ja |__| Nein</td><td>|__| Gar nicht |__| Ein wenig |__| Mittelmäßig |__| Stark |__| Sehr stark</td></tr>
</table>

Probanden-ID: ____________

4. **Möglicherweise haben Sie neben den oben genannten Erkrankungen weitere Beschwerden oder gesundheitliche Probleme. Bitte markieren Sie in der folgenden Auflistung, welche der genannten Beschwerden oder gesundheitlichen Probleme Sie haben bzw. <u>in den letzten 12 Monaten</u> hatten.**

Falls Sie in der ersten Spalte „Nein“ ankreuzen, fahren Sie bitte direkt mit dem nächsten gesundheitlichen Problem in der darunterliegenden Zeile fort. Falls Sie „Ja“ ankreuzen, geben Sie bitte an, ob Sie sich aufgrund dieses Problems in (ärztlicher) Behandlung befinden und ob dieses Problem Sie in der Ausübung Ihrer täglichen Aktivitäten (Arbeit bzw. Freizeit) beeinträchtigt. Sie haben zusätzlich die Möglichkeit, weitere Beschwerden zu ergänzen, die bislang nicht aufgeführt wurden.

**Gesundheitliches Problem**	**Haben Sie dieses gesundheitliche Problem?**	**Befinden Sie sich deshalb in (ärztlicher) Behandlung?**	**Wie stark beeinträchtigt Sie dieses gesundheitliche Problem in der Ausübung Ihrer täglichen Aktivitäten (Arbeit bzw. Freizeit)?**
Magen-Darm Probleme (z.B. häufige Bauchschmerzen, Verdauungsstörung)	|__| Ja |__| Nein	|__| Ja |__| Nein	|__| Gar nicht |__| Ein wenig |__| Mittelmäßig |__| Stark |__| Sehr stark
Gelenkschmerzen	|__| Ja |__| Nein	|__| Ja |__| Nein	|__| Gar nicht |__| Ein wenig |__| Mittelmäßig |__| Stark |__| Sehr stark
Kopfschmerzen	|__| Ja |__| Nein	|__| Ja |__| Nein	|__| Gar nicht |__| Ein wenig |__| Mittelmäßig |__| Stark |__| Sehr stark
Herz- oder Brustschmerzen	|__| Ja |__| Nein	|__| Ja |__| Nein	|__| Gar nicht |__| Ein wenig |__| Mittelmäßig |__| Stark |__| Sehr stark
Chronischer (lang andauernder) Husten	|__| Ja |__| Nein	|__| Ja |__| Nein	|__| Gar nicht |__| Ein wenig |__| Mittelmäßig |__| Stark |__| Sehr stark
Schwierigkeiten beim Atmen, Atemnot	|__| Ja |__| Nein	|__| Ja |__| Nein	|__| Gar nicht |__| Ein wenig |__| Mittelmäßig |__| Stark |__| Sehr stark
Schlafstörung	|__| Ja |__| Nein	|__| Ja |__| Nein	|__| Gar nicht |__| Ein wenig |__| Mittelmäßig |__| Stark |__| Sehr stark
Schwindelgefühl	|__| Ja |__| Nein	|__| Ja |__| Nein	|__| Gar nicht |__| Ein wenig |__| Mittelmäßig |__| Stark |__| Sehr stark
Sonstige Schmerzen oder Beschwerden, und zwar: ____________________	|__| Ja |__| Nein	|__| Ja |__| Nein	|__| Gar nicht |__| Ein wenig |__| Mittelmäßig |__| Stark |__| Sehr stark

Probanden-ID: ________________

5. **Sind Sie <u>in den letzten 12 Monaten</u> operiert worden? Gemeint ist z.B. die Entfernung der Gallenblase, der Einsatz eines künstlichen Hüftgelenks, eine Operation am Herzen, oder eine gynäkologische Operation.**

|__| Nein |__| Ja

Falls „ja" beschreiben Sie bitte kurz, welche Operationen durchgeführt wurden:

____

____

____

6. **Haben Sie <u>in den letzten 6 Monaten</u> eine Verletzung erlitten, die durch einen Unfall zuhause oder im Umfeld Ihres Hauses, durch sportliche Aktivität, durch einen Arbeitsunfall oder im Straßenverkehr verursacht wurde?**

|__| Nein |__| Ja

falls „Ja"

→ Mussten Sie deshalb ärztlich behandelt werden? |__| Ja |__| Nein

→ Hatten Sie einen Knochenbruch? |__| Ja |__| Nein

7. **Besteht bei Ihnen eine Behinderung, die vom Versorgungsamt anerkannt ist?**

|__| Nein |__| Ja

falls „Ja"

→ Welchen Grad der Schwerbehinderung haben Sie? ____________%

Probanden-ID: ________________

Allgemeine medizinische Versorgung

8. Waren Sie <u>in den letzten 6 Monaten</u> bei den folgenden Ärzten? Gemeint sind AMBULANTE Kontakte zu den Ärzten oder deren Praxispersonal (ausgenommen Behandlungen im Krankenhaus). Bitte berücksichtigen Sie hier auch die Praxisbesuche zur Abholung von Rezepten oder Überweisungen und zur Krankschreibung!

**Fachrichtung des Arztes**	**In Anspruch genommen**	**Anzahl der Kontakte <u>in den letzten 6 Monaten</u>**
Hausarzt	|__| Nein |__| Ja	_______ mal
Internist (falls nicht Ihr Hausarzt)*	|__| Nein |__| Ja	_______ mal
Diabetologe (falls nicht Ihr Hausarzt)*	|__| Nein |__| Ja	_______ mal
Kardiologe (Arzt für Herzkrankheiten)	|__| Nein |__| Ja	_______ mal
Nephrologe (Arzt für Nierenkrankheiten)	|__| Nein |__| Ja	_______ mal
Urologe	|__| Nein |__| Ja	_______ mal
Gynäkologe	|__| Nein |__| Ja	_______ mal
Orthopäde	|__| Nein |__| Ja	_______ mal
Gefäßchirurg	|__| Nein |__| Ja	_______ mal
Radiologe (Röntgenarzt)	|__| Nein |__| Ja	_______ mal
Hals-Nasen-Ohrenarzt	|__| Nein |__| Ja	_______ mal
Augenarzt	|__| Nein |__| Ja	_______ mal
Dermatologe (Hautarzt)	|__| Nein |__| Ja	_______ mal
Neurologe	|__| Nein |__| Ja	_______ mal
Arzt für Psychosomatik (keine Psychotherapie)**	|__| Nein |__| Ja	_______ mal
Psychiater (keine Psychotherapie)**	|__| Nein |__| Ja	_______ mal
Sonstiger Arzt *(bitte benennen)*:	|__| Nein |__| Ja	_______ mal
Sonstiger Arzt *(bitte benennen)*:	|__| Nein |__| Ja	_______ mal

*Falls Ihr Diabetologe bzw. Ihr Internist zugleich ihr Hausarzt ist, machen Sie bitte entsprechende Angaben nur einmal.

**Bitte nur Kontakte angeben, die einen anderen Anlass als eine Psychotherapie hatten. Psychotherapie wird unten erfragt.

Probanden-ID: ________________

9. Haben Sie <u>in den letzten 6 Monaten</u> einen Hausbesuch bestellen müssen?

|__| Ja, nämlich _______ mal |__| Nein

10. Wurden Sie <u>in den letzten 6 Monaten</u> in einem Krankenhaus AMBULANT behandelt (ausgenommen Notfallbehandlungen und Übernachtungen im Krankenhaus)?

|__| Ja, nämlich _______ mal |__| Nein

Falls „ja" beschreiben Sie bitte kurz, was dort gemacht wurde:

____

____

____

11. Haben Sie <u>in den letzten 6 Monaten</u> eine Krankenhaus-Ambulanz oder ärztlichen Notdienst/ Notarzt o.ä. <u>wegen eines Notfalls</u> aufgesucht (ausgenommen Übernachtungen im Krankenhaus)?

|__| Ja, nämlich _______ mal |__| Nein

Falls „ja" beschreiben Sie bitte kurz, was gemacht wurde:

____

____

____

Probanden-ID: _________________

12. Bitte schätzen Sie, wie viel Zeit Sie für Ihre ambulanten Arztbesuche <u>in den letzten 6 Monaten</u> aufgewendet haben. Gemeint ist die Gesamtzeit für <u>alle</u> Ihre Arztbesuche in den letzten 6 Monaten. Machen Sie bitte entsprechende Angaben in Minuten oder Stunden.

	**An- und Abfahrtzeit**	**Wartezeit**	**Behandlungszeit**
Hausarzt	|____| Min. bzw. |____| h	|____| Min. bzw. |____| h	|____| Min. bzw. |____| h
Internist (wenn nicht Ihr Hausarzt)	|____| Min. bzw. |____| h	|____| Min. bzw. |____| h	|____| Min. bzw. |____| h
Diabetologe (wenn nicht Ihr Hausarzt)	|____| Min. bzw. |____| h	|____| Min. bzw. |____| h	|____| Min. bzw. |____| h
Kardiologe (Herzarzt)	|____| Min. bzw. |____| h	|____| Min. bzw. |____| h	|____| Min. bzw. |____| h
Nephrologe (Nierenarzt)	|____| Min. bzw. |____| h	|____| Min. bzw. |____| h	|____| Min. bzw. |____| h
Urologe	|____| Min. bzw. |____| h	|____| Min. bzw. |____| h	|____| Min. bzw. |____| h
Gynäkologe	|____| Min. bzw. |____| h	|____| Min. bzw. |____| h	|____| Min. bzw. |____| h
Orthopäde	|____| Min. bzw. |____| h	|____| Min. bzw. |____| h	|____| Min. bzw. |____| h
Gefäßchirurg	|____| Min. bzw. |____| h	|____| Min. bzw. |____| h	|____| Min. bzw. |____| h
Radiologe (Röntgenarzt)	|____| Min. bzw. |____| h	|____| Min. bzw. |____| h	|____| Min. bzw. |____| h
Hals-Nasen-Ohrenarzt	|____| Min. bzw. |____| h	|____| Min. bzw. |____| h	|____| Min. bzw. |____| h
Augenarzt	|____| Min. bzw. |____| h	|____| Min. bzw. |____| h	|____| Min. bzw. |____| h
Dermatologe (Hautarzt)	|____| Min. bzw. |____| h	|____| Min. bzw. |____| h	|____| Min. bzw. |____| h
Neurologe	|____| Min. bzw. |____| h	|____| Min. bzw. |____| h	|____| Min. bzw. |____| h
Arzt für Psychosomatik	|____| Min. bzw. |____| h	|____| Min. bzw. |____| h	|____| Min. bzw. |____| h
Psychiater	|____| Min. bzw. |____| h	|____| Min. bzw. |____| h	|____| Min. bzw. |____| h
Sonstiger Arzt:	|____| Min. bzw. |____| h	|____| Min. bzw. |____| h	|____| Min. bzw. |____| h
Sonstiger Arzt:	|____| Min. bzw. |____| h	|____| Min. bzw. |____| h	|____| Min. bzw. |____| h
Ambulante Behandlung im Krankenhaus	|____| Min. bzw. |____| h	|____| Min. bzw. |____| h	|____| Min. bzw. |____| h
Notfallbehandlungen	|____| Min. bzw. |____| h	|____| Min. bzw. |____| h	|____| Min. bzw. |____| h

Probanden-ID: ________________

13. Wurden bei Ihnen die folgenden medizinischen Spezialuntersuchungen <u>in den letzten 6 Monaten</u> AMBULANT durchgeführt? Bitte alles Zutreffende ankreuzen!

|__| Nein

|__| Ja, nämlich:

**Untersuchung**	**Ja**	**Wie oft:**	**Was wurde untersucht:**
Sonographie (Ultraschalluntersuchung)	|__|	______ mal	
Röntgen	|__|	______ mal	
Spiegelung von Magen oder Darm	|__|	______ mal	
Computertomographie (CT)	|__|	______ mal	
Kernspintomographie (MRT)	|__|	______ mal	
EKG	|__|	______ mal	
Sonstiges (*bitte kurz beschreiben*):	|__|	______ mal	
Sonstiges (*bitte kurz beschreiben*):	|__|	______ mal	
Sonstiges (*bitte kurz beschreiben*):	|__|	______ mal	

Probanden-ID: ________________

14. Waren Sie in den letzten 6 Monaten in einem Krankenhaus zur STATIONÄREN Behandlung?

|__| Nein

|__| Ja, nämlich:

Name und Ort der Einrichtung	Abteilung	Aufnahmegrund bzw. Grund des Aufenthalts	Ist eine Operation durchgeführt worden?	Verweildauer
			|__| Ja<br>|__| Nein	___Tage oder<br>___Wochen
			|__| Ja<br>|__| Nein	___Tage oder<br>___Wochen
			|__| Ja<br>|__| Nein	___ Tage oder<br>___ Wochen
			|__| Ja<br>|__| Nein	___ Tage oder<br>___ Wochen
			|__| Ja<br>|__| Nein	___ Tage oder<br>___ Wochen

Probanden-ID: ________________

15. Haben Sie in den letzten 6 Monaten einen Psychotherapeuten aufgesucht?

|__| Nein

|__| Ja, nämlich:

Anzahl Kontakte	Selbstgetragene Kosten in €* (Gesamtsumme)	Gesamter Zeitaufwand in Minuten oder Stunden**
_____ mal	_______ €	|____| Min. oder |____| h

* Falls Sie den Betrag nicht genau nennen können, schätzen Sie ihn bitte.
** Falls Sie den Zeitaufwand nicht genau nennen können, schätzen Sie ihn bitte.

16. Haben Sie in den letzten 6 Monaten einen Krankengymnasten, Heilpraktiker oder andere Therapeuten aufgesucht?

|__| Nein

|__| Ja, nämlich:

Therapeut (Fachrichtung)	Anzahl der Kontakte	Leistungen (bitte kurz beschreiben)	Selbstgetragene Kosten in €* (Gesamtsumme)	Gesamter Zeitaufwand in Minuten oder Stunden**
Krankengymnast	_____ mal		_______ €	|____| Min. oder |____| h
Heilpraktiker	_____ mal		_______ €	|____| Min. oder |____| h
Sonstiger Therapeut (*bitte benennen*):	_____ mal		_______ €	|____| Min. oder |____| h
Sonstiger Therapeut (*bitte benennen*):	_____ mal		_______ €	|____| Min. oder |____| h

* Falls Sie den Betrag nicht genau nennen können, schätzen Sie ihn bitte.
** Falls Sie den Zeitaufwand nicht genau nennen können, schätzen Sie ihn bitte.

Probanden-ID: _________________

17. Haben Sie <u>in den letzten 6 Monaten</u> an Maßnahmen zur Gesundheitsförderung teilgenommen? Gemeint sind z.B. Kurse, Schulungen oder Beratungen, die sich mit Ernährung, Bewegung, Entspannung und Sport oder Fitness befassen.

|__| Nein

|__| Ja, nämlich:

Kurze Beschreibung	Selbstgetragene Kosten in €* (Gesamtsumme)	Gesamter Zeitaufwand in Minuten oder Stunden**
	________ €	|____| Min. oder<br>|____| h
	________ €	|____| Min. oder<br>|____| h
	________ €	|____| Min. oder<br>|____| h
	________ €	|____| Min. oder<br>|____| h

* Falls Sie den Betrag nicht genau nennen können, schätzen Sie ihn bitte.
** Falls Sie den Zeitaufwand nicht genau nennen können, schätzen Sie ihn bitte.

18. Welche Art der Krankenversicherung haben Sie?

|__| gesetzlich

|__| privat

|__| gesetzlich mit privater Zusatzversicherung

Probanden-ID: ________________

19. Nehmen Sie an einem *Disease-Management-Programm (DMP)* teil? Gemeint sind spezielle Programme, die von den Krankenkassen für chronisch kranke Patienten über den Hausarzt/ behandelnden Arzt angeboten werden und in die Sie sich eintragen mussten.

|__| Ja, nämlich:

|__| DMP für Diabetes

|__| anderes DMP, bitte benennen: ________________________

|__| Nein

|__| Weiß ich nicht

Behandlung des Diabetes

20. Wie häufig messen Sie gegenwärtig Ihren Blutzucker?

________ mal pro Tag

________ mal pro Woche

|__| gar nicht

21. Wie wird Ihr Diabetes gegenwärtig (d.h. in den letzten 2-4 Wochen) behandelt?

Mehrere Angaben sind möglich!

|__| Mit Diät oder Bewegung

|__| Mit blutzuckersenkenden Tabletten

|__| Mit Insulin

|__| Sonstiges (z.B. mit Spritzen von Byetta oder Victoza): ________________________

Probanden-ID: ________________

Bitte beantworten Sie <u>die Fragen 22 bis 24</u> nur wenn Sie mit Insulin behandelt werden.

22. Wie häufig spritzen Sie pro Tag Insulin?

In der Regel __________ mal pro Tag

23. Spritzen Sie Insulin in vom Arzt fest vorgegebener täglicher Menge oder haben Sie mit dem Arzt eine „flexible Therapie“ vereinbart, d.h. Sie bestimmen die Einheiten pro Mahlzeit oder Tageszeit selbständig:

|__| Insulinmenge ist fest vorgegeben

|__| Insulinmenge wird selbst nach Bedarf bestimmt

24. Haben Sie die Art der Insulin-Zuführung <u>in den letzten 6 Monaten</u> gewechselt (z.B. Wechsel von Spritze zu Pen oder Pumpe)?

|__| Nein

|__| Ja, und zwar von ___________ auf ___________ ungefähr seit _____________

<u>Gesundheit, Beruf und Alltag</u>

25. Wie waren Sie <u>in den letzten 6 Monaten</u> beruflich beschäftigt?

|__| Voll erwerbstätig (mit einer wöchentlichen Arbeitszeit von 35 Stunden und mehr)

|__| Teilzeitbeschäftigt

|__| Arbeitslos

|__| Erwerbsunfähig

|__| Rentner(in), Pensionär(in), im Vorruhestand

|__| Sonstige Tätigkeit: ____________________________

26. Waren Sie <u>in den letzten 6 Monaten</u> krankgeschrieben?

|__| Nein

|__| Ja, für insgesamt ______ Tage

Probanden-ID: ________________

27. Gab es <u>in den letzten 4 Wochen</u> Tage, an denen Sie so krank waren, dass Sie Ihren üblichen (Arbeits-)Tätigkeiten nicht nachgehen konnten? Bitte denken Sie an alle Tage, an denen Sie wegen Krankheit nicht zur Arbeit gehen konnten <u>oder</u> Ihren üblichen Tätigkeiten nicht nachgehen konnten, auch wenn Sie nicht ärztlich krankgeschrieben waren. Falls Sie die Zahl nicht wissen, schätzen Sie bitte möglichst genau!

|__| Nein
|__| Ja, nämlich _____ Tage

28. Haben Sie wegen Ihres Gesundheitszustandes <u>in den letzten 4 Wochen</u> Hilfe für Arbeiten in Anspruch nehmen müssen, die Sie üblicherweise selber erledigen (z.B. um den Haushalt oder Einkäufe zu erledigen)?

|__| Nein
|__| Ja, nämlich:

**Art der Hilfen**	**Gesamter Zeitaufwand in Stunden***	**Selbstgetragene Kosten** (Gesamtsumme)**
Hilfe von Familienangehörigen, Freunden oder Bekannten	_______ h	_______ €
Haushaltshilfen	_______ h	_______ €
Ambulante Pflegedienste (z.B. Caritas)	_______ h	_______ €
Sonstiges, nämlich: ________________________________	_______ h	_______ €

* Falls Sie den Zeitaufwand nicht genau nennen können, schätzen Sie ihn bitte.
** Falls Sie den Betrag nicht genau nennen können, schätzen Sie ihn bitte.

29. Haben Sie <u>in den letzten 6 Monaten</u> eine Erwerbsunfähigkeitsrente beantragt?

|__| Nein |__| Ja

Falls „Ja“, ist Ihr Rentenantrag schon bewilligt worden?

|__| Ja, der Bescheid gilt ab ___ ____ _____ |__| Nein

Probanden-ID: ________________

Angaben zu Ihrer Person

30. Ihr Geburtsjahr: ______________

31. Ihr Geschlecht: Männlich |__| Weiblich |__|

32. Welchen Familienstand haben Sie?

Ledig |__|

Verheiratet |__|

Geschieden |__|

Verwitwet |__|

33. Leben Sie mit einem (Ehe-)Partner/ einer (Ehe-)Partnerin zusammen?

Ja |__|

Nein |__|

34. Welche Nationalität haben Sie?

Deutsch |__|

Andere |__|, nämlich: ____________________________________

Falls „andere", wie lange wohnen Sie schon in Deutschland?

Seit _______ Jahr(en)

35. Welchen allgemeinbildenden Schulabschluss haben Sie? Bitte geben Sie nur Ihren höchsten Schulabschluss an.

Noch Schüler .... |__|

Schule beendet ohne Abschluss .... |__|

Volks-/ Hauptschulabschluss bzw. Polytechnische Oberschule (mit Abschluss der 8. oder 9. Klasse) .... |__|

Mittlere Reife, Realschulabschluss bzw. Polytechnische Oberschule (mit Abschluss der 10. Klasse) .... |__|

Fachhochschulreife (Abschluss einer Fachoberschule etc.) .... |__|

Abitur bzw. Erweiterte Oberschule mit Abschluss der 12. Klasse (Hochschulreife) .... |__|

Anderer Schulabschluss und zwar: ___________________________ .... |__|

Probanden-ID: ________________

36. Welchen beruflichen Ausbildungsabschluss haben Sie?

Mehrere Angaben sind möglich!

Betriebliche Anlernzeit, aber keine Lehre .... |__|

Lehre/ Berufsfachschulabschluss .... |__|

Meister-, Techniker- od. gleichwertiger Fachschulabschluss .... |__|

Fachhochschulabschluss .... |__|

Hochschulabschluss .... |__|

Anderen beruflichen Ausbildungsabschluss, und zwar:

____ .... |__|

Noch in beruflicher Ausbildung (Auszubildende(r), Lehrling, Berufsfachschule) .... |__|

Student(in) .... |__|

Keinen beruflichen Ausbildungsabschluss .... |__|

37. Welchen Beruf üben Sie aus bzw. haben Sie früher ausgeübt?

____

38. Wie viele Personen leben ständig in Ihrem Haushalt?

|__| Ich lebe alleine.

|__| Ich lebe nicht alleine. Außer mir leben im Haushalt _____ weitere Personen.

39. Wie hoch ist das monatliche Nettoeinkommen Ihres Haushaltes zur Zeit insgesamt? Gemeint ist dabei die Summe, die sich aus Lohn, Gehalt, Einkommen aus selbständiger Tätigkeit, Rente oder Pension ergibt. Rechnen Sie bitte auch die Einkünfte aus öffentlichen Beihilfen, Einkommen aus Vermietung und Verpachtung, Wohngeld, Kindergeld und sonstige Einkünfte hinzu.

Ordnen Sie sich bitte in eine der folgenden Kategorien ein:

unter 1.000 €	|__|	3.500 € bis unter 4.000 €	|__|
1.000 € bis unter 1.500 €	|__|	4.000 € bis unter 4.500 €	|__|
1.500 € bis unter 2.000 €	|__|	4.500 € bis unter 5.000 €	|__|
2.000 € bis unter 2.500 €	|__|	5.000 € bis unter 6.000 €	|__|
2.500 € bis unter 3.000 €	|__|	6.000 € bis unter 8.000 €	|__|
3.000 € bis unter 3.500 €	|__|	über 8.000 €	|__|

Probanden-ID: ________________

Wir bedanken uns herzlich für Ihre Mitarbeit und bitten Sie um eine kurze Bewertung des Fragebogens

War der Fragebogen einfach oder schwierig auszufüllen? Bitte kreuzen Sie die Skala entsprechend an:

Einfach auszufüllen ←——→ Schwierig auszufüllen

|__| 1 |__| 2 |__| 3 |__| 4 |__| 5 |__| 6

Wie beurteilen Sie die Länge des Fragebogens? Bitte kreuzen Sie wieder die Skala entsprechend an:

In Ordnung ←——→ Viel zu lang

|__| 1 |__| 2 |__| 3 |__| 4 |__| 5 |__| 6

Wie viel Zeit haben Sie zum Ausfüllen des Fragebogens gebraucht?

_______________ Minuten

Haben Sie noch Anmerkungen zum Fragebogen?

____

____

____

____

Herzlichen Dank!
