# Supplementary material for: Pre-test of questions on health-related resource use and expenditure, using behaviour coding and cognitive interviewing techniques
Source: BMC Health Serv Res. 2012 Sep 6;12:303. doi: 10.1186/1472-6963-12-303 (PMC3470966; doi:10.1186/1472-6963-12-303)
Supplement: Additional file 2 — Revised questionnaire (English translation). [file 1472-6963-12-303-S2.pdf]

Study Participant ID: ___________ Date: ___________ Interviewer ID: ___________

Questionnaire

on Health and Health Care

Thank you very much for participating in this survey!

Study Participant ID: ___________ Date: ___________ Interviewer ID: ___________

Diabetes, Other Illnesses and Health Issues

1. Which type of diabetes do you have?

Type 1 |__|

Type 2 (“Adult Onset Diabetes”) |__|

Other |__|

Please specify: ___________________

Don’t know |__|

2. When were you diagnosed with diabetes?

_______ years ago, or in the year: __________

_______ months ago (if the diagnosis occurred less than one year ago)

Don’t know |__|

3. On the following pages you will find a list of various medical conditions. Please indicate which of these conditions you have or have had in the last 12 months. This refers to conditions that were diagnosed by your physician.

If you check “No” in the first column, please continue directly with the next condition in the line below. If you check “Yes,” please indicate whether you receive (medical) treatment for this condition and whether this condition affects you in your activities of daily life (work and leisure time). At the end of the list you may add other conditions that were not listed.

Study Participant ID: ____________

**Medical Condition**	**Has this condition been diagnosed by your physician?**	**Are you receiving (medical) treatment for this condition?**	**How much does this condition affect you in your daily activities (work and leisure)?**
Hypertension (high blood pressure)	|__| Yes |__| No	|__| Yes |__| No	|__| Not at all |__| A little |__| Moderately |__| Severely |__| Extremely
PAD (peripheral arterial disease: Pain in the legs or calves during walking, forcing you to stop so that the pain lessens)			
Cardiac circulatory disorders (angina pectoris)			
Heart attack			
Cardiac insufficiency			
Circulatory disorders of the brain			
Stroke			
TIA (transient ischemic attack of the brain, with stroke like symptoms)			
Eye disorders (e.g. damage at the back of the eye, cataracts)			
Disorders of the leg or foot nerves (e.g. sensation of burning, tingling, numbness)			

**Medical Condition**	**Has this condition been diagnosed by your physician?**	**Are you receiving (medical) treatment for this condition?**	**How much does this condition affect you in your daily activities (work and leisure)?**
Inflammation, ulcers or wounds on the feet that heal poorly			
Amputation of the feet or legs			
Kidney disease (e.g. proteinuria)			
Renal dialysis			
Cancer (malignant tumor)			
Thyroid disorder			
Gout			
Chronic (long lasting) back pain			
Inflammatory disorders of the joints or spine (e.g. arthritis)			
Other disorders of the joints or spine			
Stomach or duodenal ulcers or chronic gastritis			
Inflammatory bowel disease (e.g. ulcerative colitis, Crohn's disease)			
Other bowel disease			

Study Participant ID: ____________

**Medical Condition**	**Has this condition been diagnosed by your physician?**	**Are you receiving (medical) treatment for this condition?**	**How much does this condition affect you in your daily activities (work and leisure)?**
Gall stones			
Frequent urinary tract infections (bladder infection)			
Chronic inflammation of the liver (hepatitis)			
Allergy or allergies, hay fever			
Bronchial asthma			
Chronic bronchitis or chronic obstructive pulmonary disease (COPD)			
Anemia			
Chronic skin disorders (e.g. neurodermatitis, psoriasis)			
Migraine			
Epilepsy			
Parkinson’s Disease			
Depression			
Other medical condition, please specify: ____________			
Other disorder, please specify: ____________			

Study Participant ID: ____________

Study Participant ID: ____________

4. In addition to the above medical conditions you may possibly have other health issues. In the following list, please mark which of the health issues you have or have had <u>in the last 12 months</u>.

If you check "No" in the first column, please continue directly with the next health issue in the line below. If you check "Yes," please indicate whether you receive (medical) treatment for this health issue and whether this issue affects you in your activities of daily life (work and leisure time). At the end of the list you may add other health issues that were not listed.

**Health issue**	**Do you have this health issue?**	**Are you receiving (medical) treatment for this health issue?**	**How much does this health issue affect you in your daily activities (work and leisure)?**
Gastrointestinal issues (e.g. frequent abdominal pain, indigestion)	|__| Yes |__| No	|__| Yes |__| No	|__| Not at all |__| A little |__| Moderately |__| Severely |__| Extremely
Joint pain			
Headache			
Heart or chest pains			
Chronic (long lasting) cough			
Difficulties breathing, breathlessness			
Sleep disorder			
Dizziness			
Other pain or health issue, please specify: ____________________			

Study Participant ID: ______________

5. **Have you had surgery <u>in the last 12 months</u>? This refers to surgeries such as gall bladder removal, insertion of an artificial hip joint, heart surgery, or gynecological surgery.**

|__| No |__| Yes

If "Yes," please provide a short description of the type of surgery that was performed:

____

____

____

6. **<u>In the last six months</u>, have you had an injury caused by an accident at home or close to home, or caused by sports, by an accident at work, or a traffic accident?**

|__| No |__| Yes

if "Yes"

→ Did you have to receive medical treatment for it? |__| Yes |__| No

→ Did you have a bone fracture? |__| Yes |__| No

7. **Do you have a disability that is recognized by the social services administration office?**

|__| No |__| Yes

if "Yes"

→ Which level of severe disability do you have? ____________ %

Study Participant ID: ______________

General Medical Care

8. In the last 6 months, have you seen any of the following physicians? This refers to OUTPATIENT contacts to these doctors or their office personnel (except for treatment in the hospital). Please also consider physician visits to obtain prescriptions or to get referrals and sick leave authorizations.

**Doctor's medical specialty**	**Visited**	**Number of contacts in the last 6 moths**
Primary physician	|__| No |__| Yes	_______ times
Specialist in internal medicine*		
Diabetologist *		
Cardiologist (physician for heart diseases)		
Nephrologist (physician for kidney diseases)		
Urologist		
Gynecologist		
Orthopedic physician		
Vascular surgeon		
Radiologist		
Ear, nose, throat specialist		
Ophthalmologist		
Dermatologist		
Neurologist		
Specialist for psychosomatic disorders (not for psychotherapy)**		
Psychiatrist (not for psychotherapy)**		
Other physician *(please specify)*:		
Other physician *(please specify)*:		

* If your diabetologist or specialist in internal medicine is you primary physician, please mention him or her only once.

** Please only indicate contacts that occurred for something other than psychotherapy. Questions on psychotherapy follow below.

Study Participant ID: ______________

9. Have you had to ask for a house call <u>in the last 6 months</u>?

|__| Yes, _______ times |__| No

10. <u>In the last 6 months</u>, have you received OUTPATIENT treatment in a hospital (except for emergency treatment and overnight hospital stays)?

|__| Yes, _______ times |__| No

If "Yes," please provide a short description of what type of treatment you received:

____

____

____

11. <u>In the last 6 months</u>, have you visited the emergency room or a medical emergency service or something similar <u>due to an emergency</u> (except for overnight hospital stays)?

|__| Yes, _______ times |__| No

If "Yes," please provide a short description of what type of treatment you received:

____

____

____

Study Participant ID: ______________

12. **Please provide an estimate of how much time you have spent on your outpatient physician visits <u>in the last six months</u>. This refers to the total time spent for <u>all</u> your doctor visits in the last six months. Please indicate the corresponding time in minutes or hours.**

	**Travel time to and from**	**Time spent waiting**	**Treatment time**
Primary physician	|____| minutes or |____| h	|____| minutes or |____| h	|____| minutes or |____| h
Internal medicine physician (if he/she is not your primary physician)			
Diabetologist (if he/she is not your primary physician)			
Cardiologist (physician for heart diseases)			
Nephrologist (physician for kidney diseases)			
Urologist			
Gynecologist			
Orthopedic physician			
Vascular surgeon			
Radiologist			
Ear, nose, throat physician			
Ophthalmologist			
Dermatologist			
Neurologist			
Specialist for psychosomatic disorders			
Psychiatrist			
Other physician:			
Other physician:			
Outpatient treatment in the hospital			
Emergency treatments			

Study Participant ID: _______________

13. <u>In the last 6 months</u>, have you had any of the following special medical tests as an OUTPATIENT? Please check all that apply.

|__| No

|__| Yes, the following:

**Test**	**Yes**	**How many times:**	**What was examined:**
Sonography (ultrasound)	|__|	_______ times	
X-ray	|__|	_______ times	
Gastroscopy or colonoscopy	|__|	_______ times	
Computer tomography (CT)	|__|	_______ times	
MRI	|__|	_______ times	
ECG	|__|	_______ times	
Other (*please describe briefly*):	|__|	_______ times	
Other (*please describe briefly*):	|__|	_______ times	
Other (*please describe briefly*):	|__|	_______ times	

Study Participant ID: _______________

14. <u>In the last 6 months</u>, have you gone to a hospital for INPATIENT treatment?

|__| No

|__| Yes, as follows:

**Name and location of the institution**	**Department**	**Reason for hospitalization or hospital stay**	**Was surgery performed?**	**Duration of hospital stay**
			|__| Yes<br>|__| No	___ days or<br>___ weeks

Study Participant ID: ______________

15. In the last 6 months, have you gone to see a psychotherapist?

|__| No

|__| Yes, as follows:

Number of contacts	Costs paid by you, in euros* (total amount)	Total time spent, in minutes or hours**
_____ times	€ _______	|____| minutes or |____| h

* If you are not able to indicate the exact amount, please provide an estimate.
** If you are not able to indicate the exact amount of time spent, please provide an estimate.

16. In the last 6 months, have you gone to see a physical therapist, naturopath, or other therapists?

|__| No

|__| Yes, as follows:

Therapist (Specialty)	Number of contacts	Services rendered (please describe briefly)	Costs paid by you, in euros* (total amount)	Total time spent, in minutes or hours**
Physical therapist	_____ times		€ _______	|____| minutes or |____| h
Naturopath				
Other Therapist (*please specify*):				
Other Therapist (*please specify*):				

* If you are not able to indicate the exact amount, please provide an estimate.
** If you are not able to indicate the exact amount of time spent, please provide an estimate.

Study Participant ID: ______________

17. <u>In the last 6 months</u>, have you participated in health improvement measures? This term refers to measures such as courses, training, or counseling relating to nutrition, exercise, stress relief, as well as sports or fitness.

|__| No

|__| Yes, the following:

Brief description	Costs paid by you, in euros* (total amount)	Total time spent, in minutes or hours**
	€ ________	|____| minutes or |____| h

* If you are not able to indicate the exact amount, please provide an estimate.
** If you are not able to indicate the exact amount of time spent, please provide an estimate.

18. What type of health insurance do you carry?

|__| statutory

|__| private

|__| statutory with private supplementary insurance

Study Participant ID: ______________

19. Are you enrolled in a *Disease Management Program (DMP)*? This term refers to special programs offered to chronically ill patients by health insurance funds via the primary physician/treating physician and for which you would have to register.

|__| Yes, the following:

|__| DMP for diabetes

|__| other DMP, please specify: _________________________

|__| No

|__| Don't know

Diabetes Treatment

20. At present, how often do you measure you blood sugar level?

________ times per day

________ times per week

|__| Not at all

21. At present (i.e. in the last two to four weeks), how is your diabetes treated?

Several answers are possible.

|__| with diet or exercise

|__| with blood sugar lowering tablets

|__| with insulin

|__|other (e.g. with injections by Byetta or Victoza): _________________________

Study Participant ID: ______________

Please only answer questions 22 to 24 if you are treated with insulin.

22. How many times per day do you inject insulin?

Normally ___________ times per day

23. Do you inject insulin at a daily amount that was predefined by your physician or have you and your physician agreed upon "flexible therapy," i.e. the units are determined by you depending on the meal or time of day:

|__| Insulin amount is predefined

|__| Insulin amount is determined by me according to need

24. In the last 6 months, have you changed the type of insulin administration (e.g. changed from syringe to pen or pump)?

|__| No

|__| Yes, from ____________ to ____________ approximately since ______________

Health, Work and Daily Life

25. In the last 6 months, what type of occupation have you had?

|__| Working fulltime (35 hours or more per week)

|__| Working part-time

|__| Unemployed

|__| Unable to work

|__| Retired or in early retirement

|__| Other type of work: ______________________________

26. In the last 6 months, have you taken sick leave?

|__| No

|__| Yes, for a total of _______ days

Study Participant ID: ______________

27. **<u>In the last four weeks</u>, were there days during which you were so ill that you could not carry out your normal (work) activities? Please consider all days during which you were unable to go to work due to illness or could not carry out your normal activities, even if your doctor did not give you sick leave authorization. If you do not know the exact number of days, please provide a close estimate.**

|__| No

|__| Yes, _____ days

28. **<u>In the last four weeks</u>, due to your health, have you had to accept help for chores that you normally carry out yourself (e.g. to do housework or run errands)?**

|__| No

|__| Yes, the following:

Type of help	Total time spent, in hours*	Costs paid by you** (total amount)
Help from family, friends or acquaintances	_______ h	€ _______
Home help	_______ h	€ _______
Home healthcare associations (e.g. Caritas)	_______ h	€ _______
Other, please specify: ______________________________________	_______ h	€ _______

* If you are not able to indicate the exact amount of time spent, please provide an estimate.
** If you are not able to indicate the exact amount, please provide an estimate.

29. **<u>In the last 6 months</u>, have you applied for disability pension?**

|__| No |__| Yes

If "Yes", has your application been approved?

|__| Yes, the decision is valid from ___ / ____ / _____ |__| No

Study Participant ID: ______________

Personal Information

30. Your year of birth: _______________

31. Your sex: Male |__| Female |__|

32. What is your marital status?

Single |__|

Married |__|

Divorced |__|

Widowed |__|

33. Do you live with a spouse or partner?

Yes |__|

No |__|

34. What is your nationality?

German |__|

Other |__|, please specify: _______________________________________

If "other", how long have you been living in Germany?

For _______ year(s)

35. What level of pre-college education do you have? Please only indicate your highest level.

Still a student .... |__|
Left school without a diploma .... |__|
Elementary school, ... |__|
Certificate of middle school or junior high school .... |__|
Advanced technical college entrance qualification (graduation from technical high school, etc.) .... |__|
High school diploma or extended comprehensive school (higher education entrance qualification) .... |__|
Other school certificate, please specify: ___________________________________ |__|

Study Participant ID: ______________

36. What type of occupational certificate do you have?

Multiple answers are possible.

Company training, but no apprenticeship .... |__|
Apprenticeship or certificate of vocational school completion .... |__|
Master, technician, or similar certificate of technical school completion .... |__|
Advanced technical college degree .... |__|
University degree .... |__|
Other occupational certificate, please specify: ____ |__|
Still in occupational training (trainee, apprentice, vocational school student) .... |__|
University student .... |__|
No occupational certificate .... |__|

37. What is your current occupation or what was your former occupation?

____

38. How many persons permanently live in your household?

|__| I live by myself.

|__| I do not live alone. In addition to me, there are _____ other persons living in my household.

39. What is the total monthly net income of your household at present? This refers to the total of wages, salaries, income from self-employment, retirement benefits, or pensions. Please also include income from government financial assistance, rental and lease income, housing allowance, child allowance, and any other income.

Within which one of the following categories do you fall:

under €1,000	|__|	€3,500 to €3,999	|__|
€1,000 to €1,499	|__|	€4,000 to €4,499	|__|
€1,500 to €1,999	|__|	€4,500 to €4,999	|__|
€2,000 to €2,499	|__|	€5,000 to €5,999	|__|
€2,500 to €2,999	|__|	€6,000 to €7,999	|__|
€3,000 to €3,499	|__|	€8,000 and over	|__|

Study Participant ID: ______________

Thank you for collaboration.
Please provide a short evaluation of this questionnaire.

Was this questionnaire simple or difficult to complete? Please place a check mark on the scale where appropriate:

Easy to complete ◄——————————————► Difficult to complete

|__|	|__|	|__|	|__|	|__|	|__|
1	2	3	4	5	6

What did you think about the length of the questionnaire? Please place a check mark on the scale where appropriate:

All right ◄——————————————► Much too long

|__|	|__|	|__|	|__|	|__|	|__|
1	2	3	4	5	6

How long did it take you to complete this questionnaire?

________________ minutes

Do you have any comments about this questionnaire?

____

____

____

____

Thank you!
